# Bilateral and bimodal cochlear implant listeners can segregate competing speech using talker sex cues, but not spatial cues

**DOI:** 10.1121/10.0003049

**Published:** 2021-01

**Authors:** Shelby Willis, Kevin Xu, Mathew Thomas, Quinton Gopen, Akira Ishiyama, John J. Galvin, Qian-Jie Fu

**Affiliations:** 1 Department of Head and Neck Surgery, David Geffen School of Medicine, University of California, Los Angeles, Los Angeles, California 90095, USA; 2 House Ear Institute, 2100 West Third Street, Los Angeles, California 90057, USA SLWillis@mednet.ucla.edu, KevinXu@mednet.ucla.edu, mathewthomas@g.ucla.edu, QGopen@mednet.ucla.edu, AIshiyama@mednet.ucla.edu, jgalvin@hei.org, qfu@mednet.ucla.edu

## Abstract

Cochlear implant (CI) users have greater difficulty perceiving talker sex and spatial cues than do normal-hearing (NH) listeners. The present study measured recognition of target sentences in the presence of two co-located or spatially separated speech maskers in NH, bilateral CI, and bimodal CI listeners; masker sex was the same as or different than the target. NH listeners demonstrated a large masking release with masker sex and/or spatial cues. For CI listeners, significant masking release was observed with masker sex cues, but not with spatial cues, at least for the spatially symmetrically placed maskers and listening task used in this study.

## 1. Introduction

Daily conversation often requires understanding communication from a specific target that may be masked by competing speech. Listeners can use various cues to segregate competing speech, such as differences in talker sex and/or spatial locations between the target and masker speech. However, the spectro-temporal degradation associated with cochlear implants (CIs) may limit access to talker sex and/or spatial cues. Masking release (MR) with talker sex cues can range from 9 to 12 dB in normal-hearing (NH) listeners (Brown *et al.,* 2010; Cullington and Zeng, 2008), but only from 1 to 5 dB in CI listeners (Cullington and Zeng, 2008; Visram *et al.,* 2012; Chen *et al.,* 2020). Theoretically, bilateral (CI in both ears) and bimodal (low-frequency acoustic hearing in one ear, CI in the other) listeners can take advantage of spatial cues to segregate target speech from maskers. Most of the benefit is due to head-shadow effects, where the signal-to-noise ratio (SNR) may be better in one ear than the other. For target speech from the front with spatially symmetrically placed speech maskers, NH listeners may use “better-ear glimpsing” to optimally select time-frequency segments in the ear with the better SNR (Hu *et al.,* 2018). However, bilateral and bimodal CI listeners do not appear to be able to consistently take advantage of spatial cues for this listening environment. This deficit may be due to differences in spectro-temporal resolution across ears, acoustic-electric amplitude mapping in the hearing device, frequency mismatch across ears, better-ear effects (where one ear has a better spectro-temporal representation than the other), and/or poor perception of inter-aural time differences (Hu *et al.,* 2018).

The ability to use spatial cues may also depend on the masker type. Steady noise is thought to produce “energetic” masking, while competing speech is thought to produce some combination of energetic, “envelope,” and “informational” masking (Brungart, 2001). NH listeners typically experience some release from masking with competing speech, relative to steady noise, possibly due to spectro-temporal glimpsing of the target. However, CI users generally experience poorer performance with dynamic maskers (e.g., gated noise, competing speech) than with steady noise, due to limited spectro-temporal resolution (Fu and Nogaki, 2005). It is unclear how this limitation may affect utilization of talker sex and/or spatial cues when segregating competing speech.

In this study, utilization of talker sex, spatial, and combined sex and spatial cues for segregation of competing speech was measured in bimodal CI, bilateral CI, and NH listeners. Previous studies have largely investigated the effects of talker sex and spatial cues on CI users' segregation of competing speech independently; relatively few have investigated the potential benefit of combined sex and spatial cues. Combined talker sex and spatial cues were predicted to offer a greater benefit than either cue alone in all listener groups.

## 2. Methods

### 2.1 Participants

Eight CI users participated in the study (one male and seven females; four bilateral CI listeners, four bimodal listeners). The mean age at testing was 61.4 yr (range = 36–71 yr), and the mean CI experience was 13.6 yr (range = 0.8–23.1 yr). For bimodal CI listeners, the mean warble-tone threshold across 250, 500, and 1000 Hz in sound field using subjects' clinically programmed hearing aids (HAs) was 53, 42, 45, and 30 dB hearing level (HL) for BM1, BM2, BM3, and BM4, respectively. Eighteen NH listeners (7 males and 11 females; mean age = 31.1 yr, range = 20–64 yr) served as experimental controls. All NH participants had pure tone thresholds <25 dB HL at all audiometric frequencies between 250 and 8000 Hz. Demographic information for CI and NH participants is shown in Table I. In compliance with ethical standards for human subjects, written informed consent was obtained from all participants before proceeding with any of the study procedures. This study was approved by the Institutional Review Board of the University of California, Los Angeles.

**Table 1.** Demographic information and mean SRTs for the segregation cue conditions for CI and NH participants. Age test = age at testing (years); CI exp = years of CI experience after implantation; BI = bilateral CI; BM = bimodal CI; AB = Advanced Bionics; AVE = average; * = hearing aid device type.

			Left ear		Right ear		Mean binaural SRT (dB TMR)			
							Segregation cue			
Subject	Gender	Age test (yr)	Device (processor)	CI exp (yr)	Device (processor)	CI exp (yr)	No masker sex/no spatial	Masker sex	Spatial	Masker sex + spatial
BI1	F	36	Cochlear (Nucleus 6)	9.1	Cochlear (Nucleus 6)	13.1	5.3	1.0	7.3	3.3
BI2	F	70	AB (Naida CI Q90)	14.1	AB (Harmony)	26.1	7.8	5.2	9.3	5.2
BI3	F	65	AB (Naida CI Q70)	6.8	AB (Naida CI Q70)	7.9	11.9	6.8	14.0	8.3
BI4	F	64	Cochlear (Nucleus 7)	2.5	Cochlear (Nucleus 7)	3.5	8.7	5.0	10.8	6.9
BM1	F	71	Resound*	—	Cochlear (Nucleus7)	2	7.0	6.0	9.8	6.7
BM2	M	58	AB (Naida CI Q90)	6.2	Phonak* (Naida Link UP)	—	7.8	6.7	8.6	3.7
BM3	F	56	Phonak* (Naida Link UP)	—	AB (Naida CI Q90)	0.8	9.3	6.7	11.0	6.0
BM4	F	71	Resound*	—	Cochlear (Nucleus 7)	1.6	5.2	2.2	7.7	4.1
CI AVE		61.4	Mean CI exp = 7.8 (across ears)				7.9	4.9	9.8	5.5
NH AVE		31.1					1.2	−7.8	−11.6	−12.0

### 2.2 Test materials and methods

The basic protocol was similar to the Listening in Spatialized Noise-sentence (LiSN-s) test from Brown *et al.* (2010). Speech reception thresholds (SRTs), defined as the target-to-masker ratio (TMR) that produced 50% correct word recognition, were adaptively measured using a paradigm similar to the coordinate response matrix (CRM) test (Brungart *et al.,* 2001; Tao *et al.,* 2018). Matrix-styled five-word test sentences (Crew *et al.,* 2015) were used to test recognition of target speech in the presence of masker speech. Target and masker sentences were generated for each test trial and consisted of words selected from each of the five 10-word categories (name, verb, number, color, and clothes). Two target keywords (randomly selected from the number and color categories) were embedded in a five-word carrier sentence uttered by the male target talker. The first word in the target sentence was always the name “*John*,” followed by randomly selected words from the remaining categories. The competing sentences were constructed of words produced by two different masking talkers; the words chosen for the masker sentences were different from each other and different from the target in each trial.

The target sentence was produced by a male talker and was always presented to the loudspeaker directly in front of the listener (0°). Two masker sentences that were different from each other and different from the target were presented to the front loudspeaker (0°; no spatial cue) or to the loudspeakers located at −90° and +90° relative to the target (spatial cue). The masker sentences were produced by two different male talkers that were also different from the target talker (no masker sex cue) or by two different female talkers (masker sex cue). Across all words, the mean fundamental frequency (*F*0) for the male target talker was 106 Hz. The mean *F*0 was 128 Hz for male masker 1 and 97 Hz for male masker 2; the mean *F*0 was 190 Hz for female masker 1 and 157 Hz for female masker 2. Note that *F*0 is only one of many acoustic characteristics that may differentiate talkers. For example, vocal tract length (VTL) may differ among and across male and female talkers and may be a stronger acoustic cue than *F*0 differences in CI listeners [e.g., El Boghdady *et al.* (2019)]. Across all possible sentences, the mean speaking rate for the target talker was 2.0 words per second (wps). During each test trial, the duration of each masker sentence was time-scaled to have the same duration as the target sentence. As there were five monosyllabic words in each target or masker sentence, the time-scaling needed to align the durations was minimal. However, there may have been small differences in the onset of each word in the sentences due to small variations in word duration across talkers; such differences varied from trial to trial depending on the words used for the random generation of the target and masker sentences. In all, four segregation cue conditions were tested: (1) no masker sex/no spatial (male maskers, co-located with target), (2) masker sex (female maskers, co-located with target), (3) spatial (male maskers, spatially separated from target), and (4) masker sex + spatial (female maskers, spatially separated from target).

During testing, the TMR was calculated between the target sentence and each of the masker sentences. The target sentence was always presented at 65 dBA. The masker sentences were first normalized to have the same long-term root-mean-square (rms) before any TMR adjustments. As such, when TMR = 0 dB, the target sentence was presented at 65 dBA, and each of the symmetrically placed masker sentences was presented at 65 dBA. Note that with the symmetrically placed masker sentences, the TMR was higher than the SNR at each ear. For example, when TMR = 0 dB, the SNR at each ear would be −3 dB for co-located target and masker sentences. To estimate the SNR for spatially separated target and masker sentences, 20 target and 40 masker sentences were randomly generated and were subsequently processed using a head related transfer function (HRTF) (Gardner and Martin, 1994). The HRTF was derived from measurements that were made every 5° in azimuth in the horizontal plane using a Realistic Optimus Pro 7 loudspeaker mounted 1.4 m from a Knowles Electronics Manikin for Acoustic Research. Using this calculation, when TMR = 0 dB, the SNR was approximately −4.2 dB when target and masker sentences were spatially separated. The poorer SNR at each ear (relative to the co-located condition) was due to the fact that the masker loudspeakers directly faced the left and right ears, while the target loudspeaker was directly in front of the listener. Note that the above HRTF-based analysis was used only for the purpose of estimating SNR when the maskers were spatially separated from the target. During actual testing, target and masker sentences were delivered to loudspeakers without any HRTFs. Because of difficulty in determining exact SNRs, all level adjustments to the masker sentences were made according to TMR, which allowed for more direct comparison across the co-located and spatially separated conditions.

During each test trial, target and masker sentences were presented at the desired TMR; the initial TMR was 10 dB. Participants were instructed to listen to the target sentence (produced by the male target talker and cued by the name “*John*”) and then click on one of the 10 response choices for each of the number and color categories; no other selections could be made from the remaining categories, which were grayed out. If both keywords were correctly identified, the TMR was reduced by 4 dB (initial step size) by increasing the level of the masker sentences; if not, the TMR was increased by 4 dB. After two reversals in TMR, the step size was reduced to 2 dB. The SRT was calculated by averaging the last six reversals in TMR. If there were fewer than six reversals within 20 trials, the test run was discarded, and another run was measured. Three test runs were completed for each listening condition, and the SRT was averaged across runs. The listening conditions and multiple runs were randomized within and across participants.

Subjects were tested while seated in a large (8 × 8 ft) sound-treated booth (IAC) and directly facing the front loudspeaker, with the two other loudspeakers located at −90° and +90° relative to the front loudspeaker. Stimuli were presented in sound field via multi-channel audio interface (MOTU 24 i/o) connected to a multi-channel amplifier (Dayton Audio MA1240a), connected to loudspeakers (Pyle PCB4BK) mounted on microphone stands and positioned at ear level. The target stimuli were presented in sound field at 65 dBA. Binaural SRTs were measured with both devices (CI + CI or CI + HA, depending on the subject); subjects were tested using their clinical settings for each device, which were not changed during testing. All stimuli were presented, and responses were collected using custom software [Angel Sound software (Fu, 2017)].

## 3. Results

**Fig. 1.**
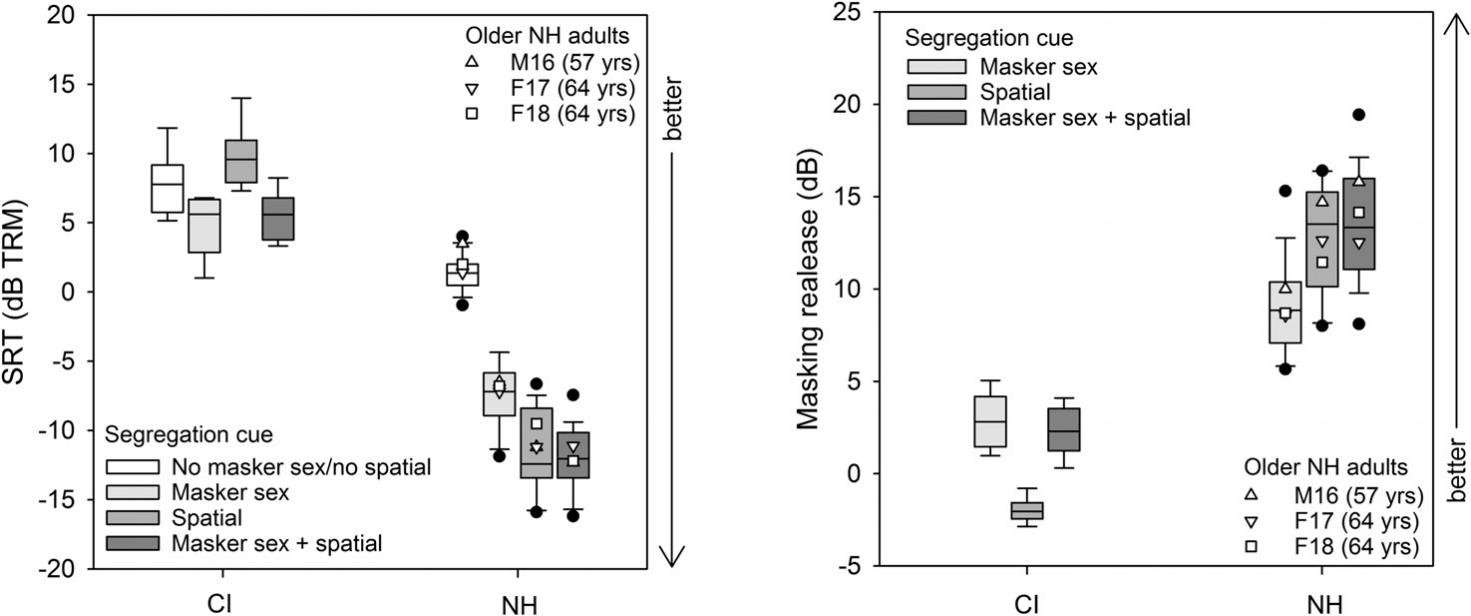
Left panel: Boxplots of SRTs for the segregation cue conditions for CI and NH listeners. Right panel: Boxplots of MR for the masker sex, spatial, and masker sex + spatial cue conditions, relative to the no masker sex/no spatial cue condition (white boxes in the left panel) for CI and NH listeners. In both panels, the horizontal solid line shows the median, the error bars show the 10th and 90th percentiles, the filled circles show outliers (>90th percentile, <10th percentile), and the open symbols show data for older NH listeners. Note that SRTs and MR are expressed in terms of TMR.

SRT data were first compared across bilateral and bimodal CI listeners. A Mann–Whitney ranked sum test found no significant difference in SRTs between bilateral and bimodal CI listeners (*U* = 110.5; *p* = 0.552). Accordingly, bilateral and bimodal CI data were combined for subsequent analyses. The left panel of Fig. 1 shows boxplots of SRTs for the four segregation cue conditions, for CI and NH listeners; mean SRTs within and across CI listeners are shown in the right columns of Table I. Note that for SRTs, higher values indicate poorer performance. In general, SRTs were lower for NH than for CI listeners. Mean SRTs for NH listeners were highest for the no masker sex/no spatial cue condition and lowest for the masker sex + spatial cue condition. In contrast, mean SRTs for CI listeners were highest for the spatial cue condition and lowest for the masker sex cue condition. An analysis of variance (ANOVA) was performed on the SRT data, with listener group (CI, NH) and segregation cue condition (no masker sex/no spatial, masker sex, spatial, masker sex + spatial) as factors. Results showed significant effects of listener group [F(1, 96) = 952.2, *p* < 0.001] and segregation cue condition [F(3, 96) = 52.1, *p* < 0.001]; there was a significant interaction [F(3, 96) = 47.0, *p* < 0.001]. *Post hoc* Bonferroni-adjusted pairwise comparisons showed that for CI listeners, SRTs were significantly poorer for the spatial cue condition than for the masker sex or masker sex + spatial cue conditions (*p* < 0.05 in both cases); there were no significant differences among the remaining segregation cue conditions (*p* > 0.05 in all cases). For NH listeners, SRTs were significantly higher for the no masker sex/no spatial cue condition than for the masker sex, spatial, and masker sex + spatial cue conditions (*p* < 0.05 in all cases) and significantly higher for the masker sex condition than for the spatial and masker sex + spatial cue conditions (*p* < 0.05); there were no significant differences among the remaining segregation cue conditions (*p* > 0.05 in all cases).

MR was calculated in terms of the dB difference when masker sex, spatial, or masker sex + spatial cues were available, relative to the no masker sex/no spatial cue condition. The right panel of Fig. 1 shows boxplots of MR for the masker sex, spatial, and masker sex + spatial cue conditions. In general, MR was larger for NH than for CI listeners. Mean MR progressively increased for NH listeners across the masker sex, spatial, and masker sex + spatial cue conditions. CI listeners experienced some MR for the masker sex and masker sex + spatial cue conditions but experienced a negative MR for the spatial cue condition. An ANOVA was performed on the MR data, with listener group (CI, NH) and segregation cue condition (masker sex, spatial, masker sex + spatial) as factors. Results showed significant effects of listener group [F(1, 72) = 865.5, *p* < 0.001] and segregation cue condition [F(2, 72) = 8.4, *p* = 0.003]; there was a significant interaction [F(2, 72) = 18.9, *p* < 0.001]. *Post hoc* Bonferroni-adjusted pairwise comparisons showed that for CI listeners, MR was significantly smaller for the spatial cue condition, relative to the masker sex and masker sex + spatial cue conditions (*p* < 0.05 in both cases); there was no significant difference between the masker sex and masker sex + spatial cue conditions (*p* > 0.05). None of the CI listeners experienced spatial MR ≥ 0 dB. A binomial test showed that spatial MR was significantly less than 0 dB (*n* = 8; *K* = 8, *p* = 0.006). For NH listeners, MR was significantly larger for the masker sex + spatial than for the masker sex cue condition (*p* < 0.05) and significantly larger for the spatial than for the masker sex cue condition (*p* < 0.05); there was no significant difference between the spatial and masker sex + spatial cue conditions (*p* > 0.05).

## 4. Discussion

NH listeners were much better able to use sex, spatial, and combined sex and spatial cues to segregate competing speech than were CI listeners. While NH listeners experienced MR for the masker sex and/or spatial cue conditions, CI listeners experienced MR only for the masker sex cue condition, with spatial cues causing SRTs to worsen. The mean MR was 9.0 dB with masker sex cues and 12.9 dB with spatial cues for NH listeners, consistent with Brown *et al.* (2010).

While significant, the MR with masker sex cues was much lower in CI listeners (2.9 dB). A negative spatial MR was observed in CI listeners (mean = −1.96 dB). Mean spatial MR was similar to that in Davis and Gifford (2018) for bilateral CI listeners (mean = −0.42), using a similar task but with only one speech masker. Gifford *et al.* (2014) reported much larger spatial MR from steady noise in bimodal and bilateral CI listeners (mean = 4.3 dB). As discussed in Sec. 2, the symmetrically placed masker sentences reduced the SNR by 1.2 dB, relative to the co-located maskers; the poorer SNR may have contributed to the negative spatial MR. The two masker sentences produced by two different talkers may have also increased the attention demands in each ear. Microphone directionality and acoustic signal compression may have further worsened the effective SNR for spatially separated maskers, relative to co-located maskers. However, using two different, symmetrically placed masker sentences may have equalized these signal processing effects across ears.

Intra- and inter-aural frequency mismatch also may have limited spatial MR. For bilateral CI users, performance would be expected to be better with the ear with less intra-aural mismatch [e.g., Svirsky *et al.* (2015)]. As such, the better ear may dominate the binaural percept and limit binaural benefit (Yoon *et al.,* 2011). For bimodal CI listeners, there is a more complex interaction between the representations at each ear. The degree of inter-aural mismatch may matter less, as perception will be dominated by acoustic hearing in the low-frequency region (where no intra-aural mismatch occurs) and by the CI in the higher frequency region (where some intra-aural mismatch might occur). Despite the very different stimulation patterns in each ear (and potentially different listening strategies for competing speech), there was no significant difference in SRTs between the present bimodal and bilateral CI listeners. This is consistent with Gifford *et al.* (2014), who found no significant difference in MR or SRTs between bilateral and bimodal CI listeners. Because of the limited number of CI participants, it is not possible to fairly compare differences in performance (or the lack thereof) between bilateral and bimodal CI listeners.

The use of two different, symmetrically placed masker sentences produced by two different talkers brings another level of complexity when considering interactions between the electric stimulation patterns. As illustrated by El Boghdady *et al.* (2019), *F*0 differences across talkers would not be expected to strongly affect the spectral envelope of the stimulation pattern, resulting in a similar TMR across talkers at different stimulation sites. However, differences in VTL strongly affected the spectral envelope across talkers, allowing for different TMRs for different spectral regions, depending on the competing talkers. Thus, VTL may allow for better glimpsing of the target in the spectral envelope of the masker. However, when the words used in the target and masker sentences are different (and when they are produced by different competing talkers), these spectral glimpsing opportunities with VTL cues may be reduced.

When both masker sex and spatial cues were available, there was no significant advantage over the dominant cue alone (masker sex for CI listeners, spatial for NH listeners). Interestingly, for CI listeners, combining the spatial cue (which caused SRTs to drop) with the masker sex cue (which caused SRTs to improve) did not worsen SRTs, relative to the masker sex cue condition. This suggests that CI listeners were able to attend to the more useful cue even when an interfering cue was available. For NH listeners, SRTs were significantly lower with the masker sex + spatial than with masker sex cue condition, but not significantly different from the spatial cue condition. It is possible that the normal inter-aural frequency match (which would promote binaural summation of the target) and the physical interactions between the target and maskers (e.g., head shadow, pinnae, etc.) contributed to the dominance of the spatial cue. Spatial cues may have saturated MR for NH listeners, allowing for little additional benefit when masker sex cues were also available.

The present findings may be limited by the small number of CI listeners and may be somewhat specific to the experimental design (CRM for competing speech with different but symmetrically placed masker sentences produced by different talkers). There was also a large age discrepancy between the present NH (mean age = 31.1 yr) and CI listeners (mean age = 61.4 yr). Age at testing has been shown to affect spatial MR in adults with binaural acoustic hearing, with significant deficits in spatial RM observed in older listeners, relative to younger listeners [4.5 dB in Besser *et al.* (2015); 7.5 dB in Zobel *et al.* (2019)]. Besser *et al.* (2015) reported that spatial MR was significantly correlated with the degree of hearing loss in the range between 6000 and 10 000 Hz (as measured with pure tone audiometry); however, Zobel *et al.* (2019) found that age at testing was significantly associated with spatial MR even after controlling for hearing loss. In the present study, all NH participants had pure tone thresholds <25 dB HL at all audiometric frequencies between 250 and 8000 Hz. The mean spatial MR was nearly identical between the three older listeners (57–64 yr old; 12.93 dB) and the 15 younger listeners (21–41 yr old; 12.87 dB). In contrast to the NH data, whether for young or older adults, the mean spatial MR was negative in CI listeners (36–71 yr old; −1.96 dB). The mean difference in spatial RM between NH and CI listeners (14.86 dB) was much greater than differences between younger and older adults reported by Besser *et al.* (2015) and Zobel *et al.* (2019). Cameron *et al.* (2011), using a test paradigm similar to the present study, showed no significant effects of age at testing for MR according to spatial or talker sex cues for NH adults between 18 and 60 yr old. Taken together, data from the present and previous studies suggest that age at testing cannot explain the large deficit in spatial RM for CI listeners, relative to NH listeners.
